# Empagliflozin improves aortic injury in obese mice by regulating fatty acid metabolism

**DOI:** 10.1515/med-2024-1012

**Published:** 2024-08-20

**Authors:** Lin Yue, Yue Wang, Cuiying Wang, Shu Niu, Xihong Dong, Yaqing Guan, Shuchun Chen

**Affiliations:** Department of Endocrinology, The Third Hospital of Shijiazhuang, Shijiazhuang, Hebei, 050000, P.R. China; Department of Ultrasonography, The Third Hospital of Shijiazhuang, Shijiazhuang, Hebei, 050000, P.R. China; Department of Endocrinology, Shijiazhuang People’s Hospital, Shijiazhuang, Hebei, 050000, P.R. China; Department of Endocrinology, Hebei General Hospital, Shijiazhuang, Hebei, 050000, P.R. China

**Keywords:** obesity, empagliflozin, fatty acids, proteomics

## Abstract

**Background:**

Empagliflozin has been shown in clinical studies to lower the risk of adverse cardiovascular events. Using proteomics, the current study aims to determine whether empagliflozin reduces aortic alterations in obese mice and to investigate its molecular mechanism of action.

**Methods:**

We constructed obese mice and then treated them with empagliflozin. Changes in the weight of the mice were recorded. Blood glucose and lipid levels were measured in each group of mice, and changes in pulse wave velocity and aortic structure were recorded. In addition, changes in aortic protein expression were detected by proteomics and analyzed bioinformatically.

**Results:**

Proteomics results showed that 507 differentially expressed proteins (DEPs) were identified in the comparison of normal and obese mice, while 90 DEPs were identified in the comparison of obese and empagliflozin-treated mice. Examination of these three groups revealed that DEPs were largely associated with the digestion of unsaturated fats. Among them, empagliflozin significantly reduced the expression of fatty acid synthase (FASN), acyl-CoA desaturase 3 (SCD3), ACSL1. and ACSL5 in the aorta of obesity-induced mice, and there was a close relationship between the four.

**Conclusion:**

Empagliflozin reduced the protein expression of FASN, SCD3, ACSL1, and ACSL5 in the aorta of obese mice and improved aortic fatty acid metabolism and reduced vascular stiffness for vasoprotective effects.

## 1 Introduction

With changing lifestyles, the prevalence of obesity is increasing and has now become a global epidemic. Obesity, hyperglycemia, dyslipidemia, hypertension, and chronic inflammatory states are often combined, increasing the chance of developing cardiovascular disease; even simple obesity without combined metabolic abnormalities has an increased prevalence of cardiovascular disease with increasing body weight [1]. There is substantial evidence that obesity-induced vascular sclerosis is an important early marker of cardiovascular disease. In obese patients, arterial stiffness is facilitated by endothelial and vascular smooth muscle cell stiffness, extracellular matrix remodeling, perivascular adipose tissue inflammation, and immune cell dysfunction. Obesity-associated insulin resistance promotes endothelial stiffness by increasing saltocorticoid receptors and activating endothelial Na+ channels, which allows impaired eNOS activation [2,3,4], dysfunctional adipose tissue near the vessel wall promotes vascular stiffness by secreting multiple vasoactive factors, and transforming growth factor and connective tissue growth factor promote extracellular matrix protein synthesis when stimulated by obesity conditions [5].

Empagliflozin is a sodium-glucose cotransport protein 2 inhibitor (SGLT2i) that works by reducing renal reabsorption of glucose to lower blood glucose levels. In addition to lowering blood glucose, the landmark EMPA-REG OUTCOME trial was the first to observe that empagliflozin also reduced the risk of major adverse cardiovascular events and all-cause mortality in patients with type 2 diabetes mellitus (T2DM) [6], and this was supported by a large number of subsequent clinical trials in which it exerted cardiovascular protective effects [7,8,9]. A number of recent basic studies have observed that empagliflozin also improves renal and cardiac function in obese mice [10,11,12], and it is unclear whether empagliflozin also has a protective effect against vascular alterations in obese patients. We hypothesize that empagliflozin similarly affects vascular injury prompted by obesity defensively. To confirm this hypothesis and further investigate the potential defense against vascular alterations by empagliflozin in obese mice, we examined the changes in structure and function of blood vessels through obese mice and observed the alterations in protein expression profile of empagliflozin on aorta of obese mice for the first time using proteomics techniques. In the present study, we wanted to clarify whether empagliflozin has a protective effect on obesity-induced aortic alterations and the possible molecular mechanisms.

## 2 Materials and methods

### 2.1 Animal experiments

Male C57BL/6 mice that were 6 weeks old were kept in typical conditions at a research facility. The mice were kept in a constant 12-h cycle of light and dark and had unrestricted access to food and drink. After a 1-week acclimatization feeding period, mice were distributed randomly into the following 2 groups: (1) control group (NCD group, *n* = 8), fed SPF-grade mice breeding diet with 34.8 kcal/100 g total calories (containing 4% fat, 20% protein, 76% carbohydrate), and (2) high-fat group (HFD group, *n* = 16), given high-level fat diet ( with 60% fat, 20% carbohydrate, 20% protein) with 524 kcal/100 g of calories. Following 12 weeks, the mice in the high-fat gathering were arbitrarily partitioned into two gatherings: (1) the high-fat group (HFD group, *n* = 8) continued to eat a high-fat diet, while the empagliflozin intervention group (Empa group, *n* = 8) fed the mice a high-fat diet and gavaged them with empagliflozin (Boehringer Ingelheim, Germany) at a dose of 10 mg/kg/day for a period of 12 weeks. The mice were anaesthetized with 2% isobarbital 45 mg/kg intraperitoneally and were left fully anaesthetized for the next step. After the experiment was over, the mice were kept on a fast overnight, blood was taken out of their eyes, clotted for 30 min at 4°C, and centrifuged for 15 min at 3,000×*g*. After blood sampling, an open chest to take out the aorta was performed and preserved frozen at −80°C. All animal experimental procedures adhered to the NIH Guide for the Care and Use of Laboratory Animals and received approval from the Animal Ethics Committee of the Hebei Provincial People’s Hospital.

### 2.2 Intraperitoneal glucose tolerance test (IPGTT)

After a 12-h fast, 2 mg/g of glucose was injected intraperitoneally into the mice. A Roche blood glucose meter was used to measure the concentration of glucose in the mice’s whole blood at 0, 15, 30, 60, 90, and 120 min after the injection of glucose.

### 2.3 Assessment of plasma levels

Insulin was measured by a mouse ELISA kit (Elite Biotech, Wuhan, China). Similarly, an ELISA kit (Nanjing Jiancheng Biotechnology Co., Ltd., Nanjing, China) was used to measure serum total cholesterol (TC), triglycerides (TG), low-density lipoprotein cholesterol (LDL-C), and high-density lipoprotein cholesterol (HDL-C). The optical thickness of the examples was recognized utilizing a completely robotized chemical marker (VERSAmax, Ameirican) and investigated utilizing SOFTmax Ace 4.3LS programming.

### 2.4 Pulse wave velocity (PWV)

PWV was measured on a high-resolution small animal sonograph (Vevo2100, Canada) to determine aortic stiffness in isoflurane-anesthetized mice (100%, oxygen flow 1.75%). The Doppler PWV is the difference in arrival time of the pressure pulse at two fixed locations along the aorta, expressed in m/s [13]. The aortic arch speed waveform was captured and then instantaneously measured at 35 mm away from the descending aorta distal that of the aortic arch.

### 2.5 Aortic pathological changes

For hematoxylin–eosin (HE) staining, Masson staining, and Elastica van Gieson (EVG) staining, approximately 3-mm-long aortic arches were fixed in 4% paraformaldehyde, dehydrated, paraffin-embedded, and serially sectioned to 4 mm thickness, respectively. The tissue measurement area was read using Image-Pro Plus 6.0 analysis software to calculate the mesothelial thickness (mesothelial area − vascular lumen area) × 2/(mesothelial circumference + vascular lumen circumference) and to calculate the aortic elastic fiber and collagen area percentages. Images were taken with an Eclipse Ci-L photomicroscope from Nikon, Japan.

### 2.6 Transmission electron microscopy (TEM)

Vascular ultrastructural changes by using Hitachi’s HT7700 transmission electron microscope from Japan were examined. Thoracic aortic rings of 1 mm^3^ size were placed in EP tubes that contained electron microscope fixative, rinsed, fixed with 1% osmium acid, dehydrated, embedded, polymerized, cut into 60–80 nm sections, and then dyed using uranyl acetate 2%/lead citrate 2.6%, followed by observation under transmission electron microscopy, and images were collected for analysis.

### 2.7 Aortic proteomics processing

After lysing mouse aortas in SDT (4% SDS, 100 mM Tris-HCl, 1 mM DTT, pH 7.6), protein concentrations were measured. After that, SDT buffer (4% SDS, 100 mM DTT, 150 mM Tris-HCl, pH 8.0) was used to treat 200 g of protein, and detergent was removed through multiple ultrafiltrations. After brooding in obscurity for 30 min, the protein suspension was processed with 4 μg trypsin at 37°C short term. Finally, the peptides that were produced were collected. Each sample of 100 µg of peptide mixture was labeled with Thermo Scientific’s Tandem Mass Tag reagent. After being attached to a C18 reversed-phase analytical column, the samples were placed in buffers A (containing 0.1% formic acid) and B (containing 84% acetonitrile and 0.1% formic acid). A data-dependent top 10 method was used to acquire mass spectrometry data, and the MASCOT engine in Proteome Discoverer 1.4 (Matrix Science, London, UK; adaptation 2.2) for ID and quantitative examination.

### 2.8 Bioinformatic analysis

Progressive grouping examination utilizes Bunch 3.0 and Java Treeview programming. Differentially expressed proteins (DEPs) were annotated with terms from the Gene Ontology (GO) using InterProScan and the NCBI BLAST + client software. Protein pathways were constructed using the database from the Kyoto Encyclopedia of Genes and Genomes (KEGG).

### 2.9 Statistical analyses

The standard error of the mean is used to display the data. The significance level was deemed to be *P* < 0.05. GraphPad Prism 8.0 was used for all of the statistical analyses. Dunnett’s test was used in conjunction with one-way analysis of variance to analyze the multiple comparisons.


**Ethical approval:** All aspects of animal care and the experimental protocols to which they were subjected to were approved by the Animal Ethics Committee of Hebei General Hospital (date March 11, 2022/No. 202285) and conducted in accordance with the Guide for the Care and Use of Laboratory Animals, published by the US National Institutes of Health (NIH Publication No. 85-23, revised 1996). All efforts were made to minimize animal suffering.

## 3 Results

### 3.1 Empagliflozin lowers body weight and helps lipid and blood glucose levels of mice

**Figure 1:**
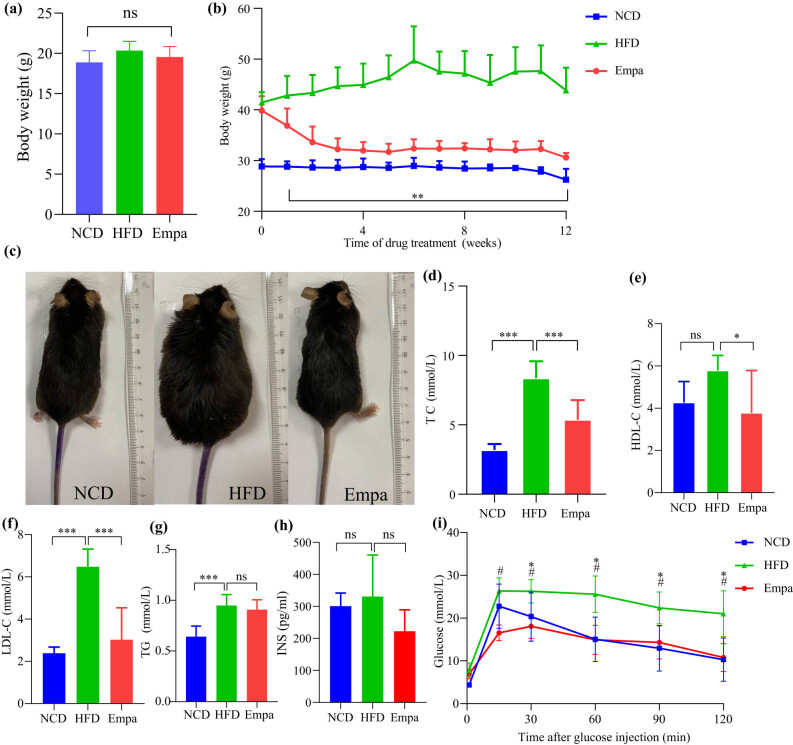
Effects of empagliflozin on body weight, serum lipids, and glucose in obese mice. (a) Body weights of the three groups of mice at the beginning of the experiment. (b) Effect of empagliflozin on body weight in three groups of mice over the course of a 12-week intervention. (c) Representative images of the three groups of mice at the end of the experiment. (d) TC. (e) HDL-C. (f) LDL-C. (g) TG. (h) INS. NS, *P* > 0.05. *P < 0.05. ***P < 0.001. (i) IPGTT. ^#^HFD vs Empa, *P* < 0.05. *HFD vs NCD, *P* < 0.05. Abbreviations: NCD, normal chow diet; HFD, high-fat diet; Sema, high-fat diet + empagliflozin; TC, total cholesterol; TG, total cholesterol; LDL-C, low-density lipoprotein cholesterol; HDL-C, high-density lipoprotein cholesterol; INS, fasting plasma insulin; IPGTT, intraperitoneal glucose tolerance test.

At the beginning of the experiment (Figure 1a), the body weight among the three groups did not differ significantly. However, following 12 weeks duration of intervention with high-fat diet, the body weight of mice belonging to the HFD group was significantly higher than that of the NCD group, whereas the body weight of mice in the HFD group was significantly lower after empagliflozin intervention (Figure 1b). A representative picture of the mice at the end of the trial also showed that empagliflozin had a significant effect on weight reduction in high-fat-fed mice (Figure 1c). We also measured lipid levels, and compared with the NCD group, high-fat-fed mice had elevated serum levels of TC, TG, and LDL-C. Empagliflozin treatment for 12 weeks reduced TC and LDL-C levels but did not significantly change TG (Figure 1d–g). IPGTT results showed that high-fat diet elevated fasting blood glucose in mice, but did not meet the diagnostic criteria for diabetes mellitus. The treatment of empagliflozin caused a trend of decrease in fasting blood glucose. After glucose stimulation, individual blood glucose in the Empa group was lower than that in the HFD group, suggesting that empagliflozin significantly lowered postprandial blood glucose, but there was no significant change in fasting insulin levels in the three groups. (Figure 1h and i).

### 3.2 Empagliflozin ameliorates structural changes in the aorta of obese mice

**Figure 2:**
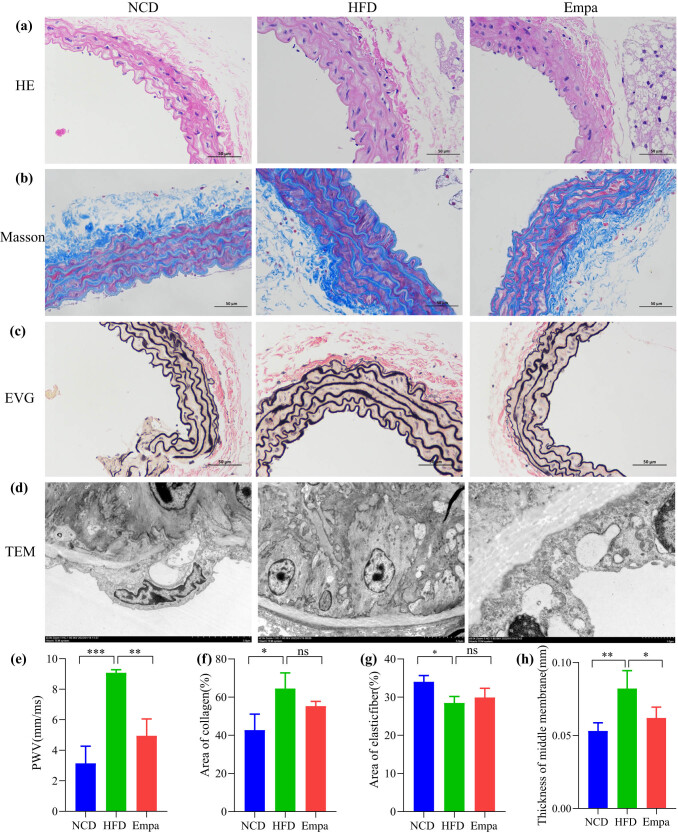
Effects of empagliflozin on the structure and function of the aorta. (a) HE. (b) Masson. (c) EVG. (d) TEM. (e) PWV. (f) Area of collagen. (g) Area of elastic fiber. (h) Thickness of middle membrane. *N* = 3. NS, *P* > 0.05. *P < 0.05. **P < 0.01. ***P < 0.001. Abbreviations: NCD, normal chow diet; HFD, high-fat diet; Sema, high-fat diet + empagliflozin; HE, hematoxylin–eosin; EVG, Elastica van Gieson; TEM, transmission electron microscopy; PWV, pulse wave velocity.

The vessel wall thickness and collagen content of mice in the HFD group were significantly higher than those in the NCD group, and after empagliflozin treatment, the thickness of the vessel wall and collagen content of the Empa group decreased (Figure 2a and b). The results of structural changes in the aorta under electron microscopy showed that disorganized elastic fibers, damaged endothelial cells and disappeared tight junctions between cells, swollen smooth muscle cells, disorganized myofilaments, and swollen intracytoplasmic organelles could be seen in the aortic tissue of mice in the HFD group, and empagliflozin treatment protected the vessels from the changes observed in the HFD group (Figure 2c and d). Mice in the HFD group exhibited higher aortic PWV compared to the NCD group (*P* < 0.05), indicating aortic stiffness, which decreased with empagliflozin application (*P* < 0.05), suggesting that empagliflozin treatment improved aortic stiffness (Figure 2e). Calculations of collagen content, elastic fiber area, and intima-media thickness were provided to show that a high-fat diet increased aortic collagen deposition, decreased elastic fiber area, and increased intima-media thickness (*P* < 0.05), while empagliflozin treatment decreased collagen deposition and increased elastic fiber area but was not statistically significant (*P* > 0.05), in contrast empagliflozin significantly reduced obesity-induced intima-media thickening (Figure 2f–h).

### 3.3 DEPs

**Figure 3:**
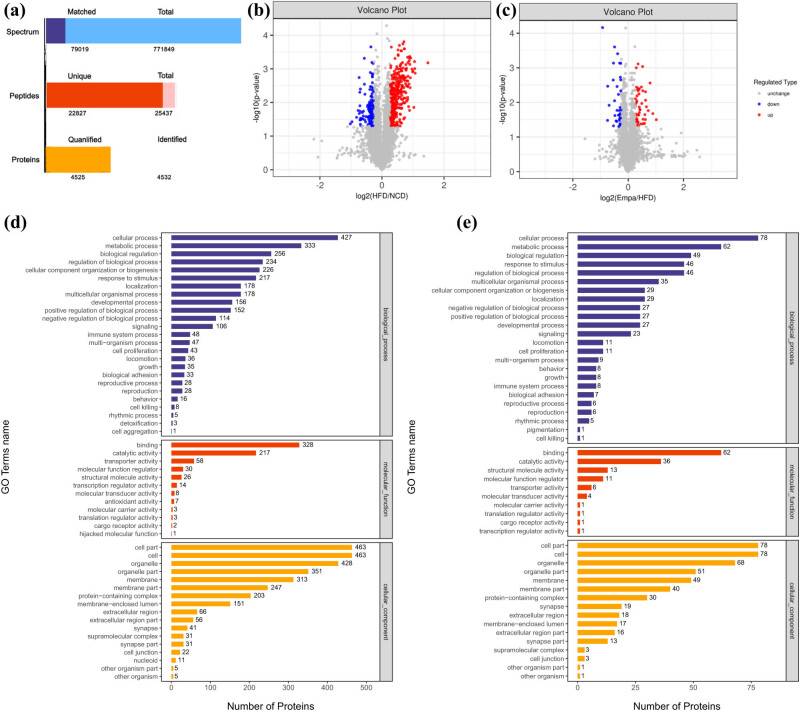
Differentially expressed proteins and GO enrichment functional analysis. (a) Identification and quantification results by LC-MS/MS mass spectrometry. (b) Volcano map of DEPs in the HFD/NCD group. (c) Volcano map of DEPs in the Empa/HFD group. (d) GO functional enrichment results of DEPs compared in the HFD/NCD group. (e) GO functional enrichment results of DEPs compared in the Empa/HFD group. Abbreviations: NCD, normal chow diet; HFD, high-fat diet; Sema, high-fat diet + empagliflozin; GO, Gene Ontology; DEPs, differentially expressed proteins.

Liquid chromatography–tandem mass spectrometry (LC–MS/MS) analysis yielded 79,019 matched spectrums out of a total of 771,849; 22,827 distinct peptides were among the 25,437 identified; 4,532 proteins were distinguished, of which 4,525 measured proteins (Figure 3a). Up-directed DEPs were characterized as *P*-value <0.05 and overlay change (FC) >1.2, and down-controlled as *P*-value <0.05 and FC <0.83. A total of 358 proteins were up-directed and 149 proteins were down-managed in the HFD bunch contrasted with the NCD bunch, while 51 DEPs were up-controlled and 39 were down-managed in the Empa bunch contrasted with the HFD bunch (Figure 3b and c). Differences in proteins between groups are depicted in volcano plots, where blue color denotes obvious downshifts, red color means obvious upshifts, and gray color denotes proteins with no differences.

### 3.4 Identification of the functions of DEPs

GO annotation was performed using Blast2Go (https://www.blast2go.com/) [14] software for all DEPs, including cellular component, molecular function, and biological process (Figure 3d and e). The DEPs were mainly located in the mitochondria in the NCD/HFD group comparison, where activity was the most enriched term in molecular function enrichment, and with respect to biological processes, DEPs were significantly enriched in mitochondrial respiratory electron chain and ATP synthesis. In contrast, in the HFD/Empa group comparison, DEPs were mainly located in the cytoplasm, performing the main molecular functions of activity and binding, and the main biological processes involved were fatty oxoacid and organic acid metabolic.

**Figure 4:**
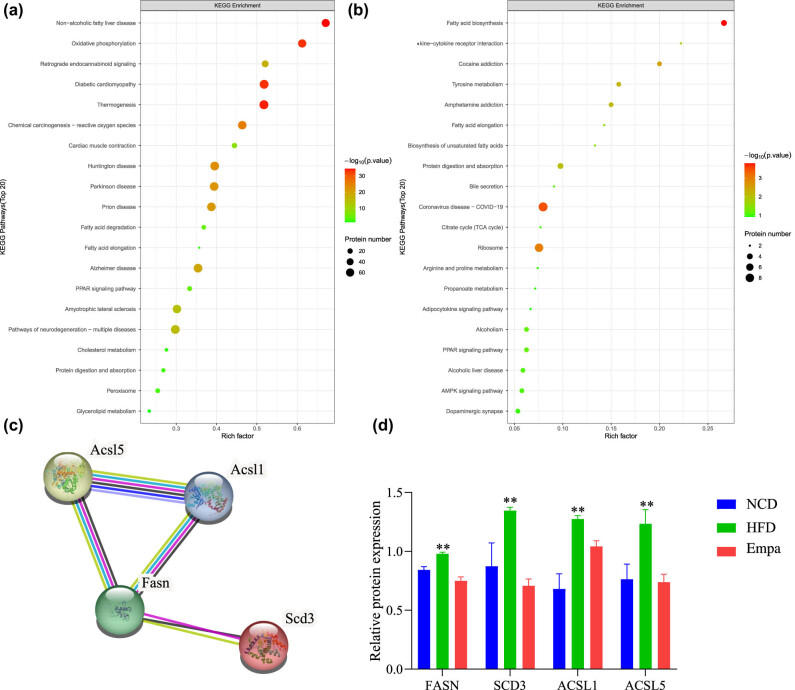
KEGG enrichment analysis and expression changes of differentially expressed proteins. (a) KEGG enrichment analysis results of DEPs compared in the HFD/NCD group. (b) KEGG enrichment analysis results of DEPs compared in the Empa/HFD group. (c) Interactions between DEPs. (d) The relative expression of DEPs between the three groups. *N* = 3. **HFD vs NCD and HFD vs Empa, *P* < 0.01. Abbreviations: NCD, normal chow diet; HFD, high-fat diet; Sema, high-fat diet + empagliflozin; KEGG, Kyoto Encyclopedia of Genes and Genomes; DEPs, differentially expressed proteins.

To further understand the pathways involved in the aortic protein changes by empagliflozin in obese mice, the DEPs were parsed and annotated using the KEGG pathway database. DEPs in the NCD/HFD group were significantly enriched in oxidative phosphorylation, diabetic cardiomyopathy, thermogenesis, and fatty acid metabolic (Figure 4a), whereas DEPs in the HFD/Empa group were mainly enriched in coronavirus disease (COVID-19), fatty acid metabolic, and ribosome (Figure 4b). Further synthesizing the above results, we found that fatty acid synthase (FASN), acyl-CoA desaturase 3 (SCD3), ACSL1, and ACSL5 were involved in fatty acid metabolic processes in both comparison groups and were closely linked to each other (Figure 4c), and their expression was significantly decreased after empagliflozin intervention (*P* < 0.01) (Figure 4d).

## 4 Discussion

In the present study, we found a protective effect of empagliflozin against obesity-induced structural and functional damage to the aorta. Although numerous clinical trials have demonstrated a cardiovascular protective effect of SGLT2-i in patients with T2DM, the potential role and mechanisms of vascular alterations due to obesity are not fully understood, and analysis of aortic proteomics could provide valuable insights, so we investigated the alterations in the aorta of high-fat-induced obese mice and the efficacy of empagliflozin after administration. Ozcelik et al. found that oral administration of empagliflozin in T2DM patients improved glycated hemoglobin, body weight, and blood pressure, and in agreement with this study, we also observed in our experiments that empagliflozin reduced body weight, lowered postprandial glucose, and improved serum cholesterol and LDL-C levels. The effect of SGLT2i on lipids in animal experiments seems to be inconclusive. Empagliflozin reduced serum cholesterol and LDL-C levels in our results, whereas most clinical studies using SGLT-2i observed a moderate increase in LDL-C levels, which was considered to be related to a decrease in LDL-C clearance from the circulation [15]. In keeping with the results of the Fadini study on the effects of empagliflozin on HDL-C levels and function [16], we did not find changes in HDL-C due to empagliflozin, and in terms of TG, our data are consistent with the results of Rau et al. that the effect of empagliflozin on TG was not significant [17].

In the proteomic results, the differential proteins FASN, SCD3, long-chain-fatty-acid-CoA ligase 1 (ACSL1), and long-chain-fatty- acid-CoA ligase 5 (ACSL5) are all involved in fatty acid metabolism. Therefore, we hypothesized that fatty acid metabolism might be related to the protective effect of empagliflozin against aortic alterations in obese mice. Studies have confirmed that the aorta of humans and a variety of animals can synthesize fatty acids from scratch as well as prolong them and that local fatty acid biosynthesis in the human aorta can promote the accumulation of aortic lipids and increase the risk of atherosclerosis [18,19]. Previous studies have shown that empagliflozin treatment protects against cardiac mitochondrial fatty acid oxidation under conditions of chronic lipid overload and protects against structural and functional cardiac damage due to disorders of lipid metabolism [20,21]. Therefore, we selected several proteins associated with fatty acid metabolism and significantly altered in an attempt to reveal the mechanism by which ampagliflozin exerts a protective effect on the aorta of obese mice.

In the aorta, exogenous uptake and de novo synthesis of fatty acids can be used for fatty acid β-oxidation, which is not important for maintaining vascular redox homeostasis but will use the carbon derived in this process to synthesize deoxyribonucleotides and maintain endothelial cell proliferation during vascular germination [22,23]. Vascular smooth muscle cells in the aorta are an important site for fatty acid β-oxidation, which can provide more energy for vascular smooth muscle cell proliferation and migration [24,25]. Proteomic results showed that empagliflozin decreased the expression of FASN, SCD3, ACSL1, and ACSL5 during adductor fatty acid synthesis and fatty acid β-oxidation. Adductor fat synthesis uses carboxylation of acetyl coenzyme A (CoA) to malonyl CoA, synthesis of long-chain fatty acids by FASN, fatty acid chain lengthening by SCD3, and esterification by long-chain acyl coenzyme A synthase (ACSL) to biologically active acyl coenzyme A, which participates in fatty acid β-oxidation. During this process, endothelial cells regulate malonyl CoA levels via FASN, and inhibition of FASN leads to post-translational malonylation of mTOR, which reduces mTOR activity and fatty acid β-oxidation [26]. Depending on the substrate, ACSL affects fatty acid metabolism in different tissues, leading to fatty liver, atherosclerosis, and diabetes [27]. ACSL1 and ACSL5 (endoplasmic reticulum) also partition fatty acids into TG synthesis [28]. Among them, ACSL5 also shuttles acyl-coenzyme A into organelles for β-oxidation [27]. In high-fat-induced obese mice, elevated expression of ACSL1 and ACSL5 increased aortic TG synthesis, increased activation and β-oxidation of free fatty acids, and promoted proliferation of endothelial cells and smooth muscle cells, while empagliflozin application decreased FASN and SCD3 during ab initio lipid synthesis, resulting in a decrease in long-chain fatty acids as substrates for fatty acid β-oxidation; decreased ACSL1 and ACSL5, reduced activated fatty acids, decreased fatty acid β-oxidation levels, and delayed the progression of arterial lesions.

SGLT2i is known to reduce cardiovascular risk in patients with T2DM; however, the potential role and mechanism of SGLT2i in the vasculature of patients without diabetes is not fully understood. Therefore, we observed the effects of empagliflozin on the aorta of obese mice, as well as applied proteomics techniques to explore the targets of empagliflozin in terms of molecular mechanisms of action. However, there are some limitations throughout the experiment: (1) the molecular mechanism of the protective effect of empagliflozin on the aorta needs to be clarified by cellular experiments; (2) the treatment duration of this experiment is relatively short, and long-term therapeutic studies of empagliflozin are needed in the future; and (3) finally, this study is an animal experiment, and its protective application in vascular alterations in obese people needs further clinical trials.

## 5 Conclusion

Empagliflozin reduces body weight, improves glucolipid metabolism, and protects aortic function in high-fat diet-induced obese mouse models. Proteomic results showed that empagliflozin decreased the expression levels of the proteins FASN, SCD3, ACSL1, and ACSL5 associated with fatty acid de novo synthesis and β-oxidation in the aorta, which may be the molecular mechanism of its vascular protective effect.
