# Corrigendum to “Empagliflozin improves aortic injury in obese mice by regulating fatty acid metabolism”

**DOI:** 10.1515/med-2025-9999

**Published:** 2025-02-18

**Authors:** Lin Yue, Yue Wang, Cuiying Wang, Shu Niu, Xihong Dong, Yaqing Guan, Shuchun Chen

**Affiliations:** Department of Endocrinology, The Third Hospital of Shijiazhuang, Shijiazhuang, Hebei, 050000, P.R. China; Department of Ultrasonography, The Third Hospital of Shijiazhuang, Shijiazhuang, Hebei, 050000, P.R. China; Department of Endocrinology, Shijiazhuang People’s Hospital, Shijiazhuang, Hebei, 050000, P.R. China; Department of Endocrinology, Hebei General Hospital, Shijiazhuang, Hebei, 050000, P.R. China

In the published article Yue L, Wang Y, Wang C, Niu S, Dong X, Guan Y, Chen S. Empagliflozin improves aortic injury in obese mice by regulating fatty acid metabolism. Open Med. (Wars) 2024; 19(1): 20241012, doi: 10.1515/med-2024-1012. PMID: 39176252 PMCID: PMC11340858, the affiliation of Lin Yue is not complete.

The correct author affiliation is as follows:

Department of Endocrinology, The Third Hospital of Shijiazhuang, Shijiazhuang, China; Department of Endocrinology, Hebei General Hospital, Shijiazhuang, China.

